# Self-Disgust Is Associated With Loneliness, Mental Health Difficulties, and Eye-Gaze Avoidance in War Veterans With PTSD

**DOI:** 10.3389/fpsyg.2020.559883

**Published:** 2020-10-30

**Authors:** Antonia Ypsilanti, Richard Gettings, Lambros Lazuras, Anna Robson, Philip A. Powell, Paul G. Overton

**Affiliations:** ^1^Department of Psychology, Sociology and Politics, Sheffield Hallam University, Sheffield, United Kingdom; ^2^School of Health and Related Research (ScHARR), The University of Sheffield, Sheffield, United Kingdom; ^3^Department of Psychology, The University of Sheffield, Sheffield, United Kingdom

**Keywords:** loneliness, self-disgust, war veterans, PTSD, depression, anxiety

## Abstract

In the present study, we examined, for the first time, the association between self-disgust, loneliness, and mental health difficulties in war veterans diagnosed with PTSD. For this purpose, we used a mixed methods design, incorporating surveys and a novel eye-tracking paradigm, and compared the findings from the PTSD veteran group (*n* = 19) to those from a general population group (*n* = 22). Our results showed that the PTSD veteran group reported almost three times higher scores in self-disgust, and significantly higher scores in loneliness and mental health difficulties (anxiety and depression), compared to the general population. Furthermore, self-disgust mediated the association between loneliness and anxiety symptoms in both groups. The results from the eye-tracking paradigm further showed that veterans with PTSD displayed a self-avoidance gaze pattern, by looking significantly more toward pictures of faces of unknown others and away from their own face—a pattern that was not replicated in the general population group. Higher self-disgust scores were significantly associated with longer total gaze to the pictures of others (vs. the self). Our findings have implications for the role of self-disgust in the mental health of war veterans.

## Introduction

Posttraumatic stress disorder (PTSD) can be conceptualized as a cluster of symptoms resulting from extremely traumatic life-threatening experiences, for example, war experiences (Sher, 2005). Diagnosing PTSD requires symptoms to have developed after the traumatic experience and the prevalence of PTSD among war veterans in the United Kingdom ranges between 4% and 12%, and varies from 2% to17% worldwide (e.g., Hotopf et al., 2006; Lee et al.). If the war stressor is chronic and especially if cruelty is involved (i.e., when war captivity and/or maltreatment are involved), the prevalence of PTSD among survivor veterans can be as high as 80–90% (Kramer et al., 1994; Tanielian et al., 2008).

Loneliness (i.e., the subjective feeling of lack of meaningful social relationships) is prevalent among war veterans (Kuwert et al., 2014; Solomon et al., 2015). Research has indicated that 44% of war veterans experience feelings of loneliness at least some of the time, and 10.4% reported often feeling lonely (Kuwert et al., 2014). Solomon and Dekel (2008) found that increased levels of PTSD among war veterans were positively associated with higher levels of self-reported loneliness. More recent studies in Israeli veterans have also shown that loneliness was cross-sectionally and longitudinally associated with posttraumatic stress reaction (Solomon et al., 2015) and posttraumatic growth (Stein et al., 2018). Combat stress seems to contribute to the development and maintenance of loneliness across time (Solomon et al., 2015) for two reasons; firstly, on the battlefield, soldiers that are exposed to trauma feel alone and defenseless, and secondly, during the homecoming period war veterans feel estranged and isolated from society because they feel that others cannot understand them (Solomon et al., 2015). As a result, they tend to report higher levels of social avoidance (Galovski and Lyons, 2004) and psychosocial difficulties (Pietrzak et al., 2010), less social support, poorer social functioning and lower life satisfaction (Tsai et al., 2012) compared to non-PTSD veterans.

Posttraumatic stress disorder is also highly comorbid with depression (Blanchard et al., 1998), anxiety (Spinhoven et al., 2014), suicidality (Kramer et al., 1994; Oquendo et al., 2003, 2005; Sher, 2005) and bipolar disorder (Dilsaver et al., 2007). In war veterans, the triple comorbidity of PTSD with anxiety and depression ranges between 11–67% (Brady and Clary, 2003; Hashemian et al., 2006; Ginzburg et al., 2010). Although there are arguments suggesting that this triple comorbidity may be the result of measurement artifacts and symptoms overlap (e.g., Franklin and Zimmerman, 2001) or shared risk factors (e.g., Hoven et al., 2005; Schumm et al., 2006; Vinck et al., 2007), longitudinal data suggests otherwise. Specifically, it has been shown that PTSD symptoms are more stable across time, and that symptoms of depression and anxiety are developed later than PTSD (Ginzburg et al., 2010).

### Self-Disgust and PTSD

Self-disgust is a negative self-conscious emotional schema that originates from the basic emotion of disgust and is directed toward physical (physical self-disgust; e.g., “I find myself repulsive”) or behavioral aspects of the self (behavioral self-disgust; e.g., “I often do things I find revolting”; Overton et al., 2008). Over the last decade, research has shown that self-disgust has been associated with a range of psychological difficulties, including social anxiety (Amir et al., 2010 insomnia (Ypsilanti et al., 2018), impaired body image and disordered eating behavior (Espeset et al., 2012; Olatunji et al., 2015), and PTSD symptoms in women with a history of sexual assault (for a recent review see Clarke et al., 2019). The role of self-disgust in such traumatic experiences in females has been related to mental contamination (Badour et al., 2013), a state that is experienced predominately in relation to moral rather than physical violations. In this case, the victims report feeling “dirty inside” and demonstrate higher self-directed disgust (Coughtrey et al., 2012).

Several studies have also highlighted the mediating properties of self-disgust on the association of cognitive processes and socio-emotional experiences with mental health outcomes. In particular, self-disgust has been found to mediate the association between dysfunctional thoughts and depression (Powell et al., 2013); loneliness and depressive symptoms in the general population (Ypsilanti et al., 2019); loneliness and anxiety symptoms in older adults (Ypsilanti et al., 2020); and PTSD symptoms (PCL-5 sub-clusters of avoidance, re-experiencing, changes in cognition and affect) and suicide risk (Brake et al., 2017) in undergraduate students. More specifically, self-disgust significantly mediated the relationship between all the sub-clusters of PCL-5 outlined in the DSM-IV-TR (American Psychiatric Association [APA], 2000) and suicide attempts. However, thus far no study has examined self-disgust in war veterans diagnosed with PTSD and its role in the development of loneliness and mental health outcomes.

### Eye-Tracking, Attentional Avoidance, and Self-Disgust

In anxiety disorders, one of the most common ways to measure attentional vigilance and disengagement to threatening stimuli is to use reaction time (RT) tasks (see Armstrong and Olatunji, 2012 for a meta-analysis). In these tasks, participants are briefly exposed to threatening stimuli (e.g., 500ms), and are asked to respond to these by pressing a button (e.g., Bar-Haim et al., 2007). According to the vigilance hypothesis, individuals with anxiety disorders show hypersensitivity to threat and, therefore, respond faster to threatening stimuli compared to non-anxious individuals (Wiens et al., 2008). Alternately, the maintenance hypothesis suggests that participants exhibit difficulty disengaging from threatening stimuli such as sad pictures, which takes place after threat detection and is commonly observed in individuals with depression (Fox et al., 2001). However, RT tasks are less able to detect attentional avoidance in affective disorders, which may come into play at later stages of stimulus exposure (Cisler and Koster, 2010). Attentional avoidance is more voluntary and strategic and less automatic; therefore, it requires continuous and prolonged tasks of visual attention. (Armstrong and Olatunji, 2012). Eye-tacking methodology provides a unique opportunity to study vigilance and avoidance in affective disorders as it allows individuals to naturally dwell on the dysphoric stimuli for longer exposure times. In this context, vigilance is conceptualized as initial orientation of eye gaze to the stimulus (time to first fixation within the first second of exposure) and avoidance comes later because it requires a more conscious, voluntary evaluation of the dysphoric stimulus (Mogg et al., 1987). In other words, the individual strategically “*decides*” to avoid directing attention to the threatening stimulus.

Qualitative studies have shown that people who experience high self-disgust report that they avoid looking at their own reflections on the mirror: “I’ll suddenly, um, feel quite disgusted, possibly by my appearance, or you know, when I look in the mirror or happen to see myself in a reflective surface” and “I’ve got a lot of friends that, you know, like every time they go past…a shop window or something, they’re like…whereas…I’ll do anything to like avert my gaze” (Powell et al., 2014, p.568). An eye-tracking paradigm could allow for the assessment of attentional avoidance mechanisms in self-disgust. Moreover, people with higher levels of self-disgust may display increased attentional vigilance to others because they tend to perceive themselves as social “contaminants”: *“*What am I doing with all these people and just making them feel like there’s something horrible in the room. I should just go home and out of the way and stop making them look at me” (Powell et al., 2014, p. 571). In support of this argument, a recent eye-tracking study (Ypsilanti et al., 2020) found that older adults with higher levels of self-disgust displayed attentional avoidance to images of their own face, compared to the faces of unknown others. Based on this evidence, it is plausible that individuals with PTSD may display different responses to the faces of other people compared to when looking at their own image particularly in later stages of stimulus exposure.

### The Present Study

Previous research has examined the association between self-disgust and PTSD symptoms among females who had experienced sexual assault (Badour et al., 2012, 2013, 2014), but no study, so far, has examined the association of self-disgust with mental health difficulties among war veterans suffering from PTSD. Given that self-disgust is present in PTSD and trauma-related conditions that are not specifically pertinent to sexual assault (see Clarke et al., 2019) and that war veterans tend to experience greater mental health difficulties than the general population (Pietrzak et al., 2010; Tsai et al., 2012), it is theoretically plausible that self-disgust will be higher among war veterans with PTSD than in non-veterans and individuals without PTSD.

Furthermore, previous research has suggested that self-disgust mediated the association of loneliness with both depression and anxiety symptoms in the general population (Ypsilanti et al., 2019, 2020). It is possible that this effect can be extended to the study of PTSD among war veterans for the following reasons. Firstly, loneliness is highly prevalent among war veterans (Solomon and Dekel, 2008), and PTSD is highly comorbid with depression (Brady and Clary, 2003; Hashemian et al., 2006; Ginzburg et al., 2010), but the process that links loneliness and depression in this population remains unclear. Secondly, if loneliness contributes to the development of depressive and anxiety symptoms through self-disgust (Ypsilanti, 2018; Ypsilanti et al., 2019, 2020), then it is plausible that self-disgust may potentially explain the association of loneliness with anxiety and depression symptoms in war veterans with PTSD.

On the basis of these arguments, in the present study we hypothesize that war veterans with PTSD will exhibit higher levels of loneliness, self-disgust, and symptoms of depression and anxiety compared to a sample of the general population without PTSD history (Hypothesis 1). We also hypothesized that self-disgust would mediate the relationship between loneliness and symptoms of anxiety and depression in veterans with PTSD (Hypothesis 2). In order to further explore the role of self-disgust in mental health outcomes in war veterans with PTSD we will use a novel task to measure attentional vigilance and maintenance with an eye-tracking paradigm. Based on the “avoidance hypothesis” suggested by Powell et al. (2014) we anticipate that participants with PTSD will avoid looking at pictures of their own face compared to pictures of faces of unknown others (Hypothesis 3; Ypsilanti et al., 2020). Finally, we hypothesize that there will be a correlation between self-disgust scores and the relative difference of total eye gaze duration for self over eye gaze duration for others (Hypothesis 4).

## Materials and Methods

### Participants

Forty-two participants were recruited. Nineteen PTSD-diagnosed male veterans from the HM Armed Forces, aged between 24 and 64 years (*M* = 47.84, *SD* = 9.09) and 22 participants without PTSD history were selected from the general population, aged between 20 and 66 years (*M* = 45.40, *SD* = 13.17, males = 43.5%)—henceforth, referred to as non-PTSD participants. Power analysis was conducted with G^∗^Power (v.3.0.10) to determine the sample size required to detect significant effects in the eye-tracking task and the regression analyses (*p* < 0.05). For the eye-tracking paradigm, we used the effect sizes reported in related published research on eye gaze in high and low self-disgust groups (i.e., Ypsilanti et al., 2020). Based on those parameters (Cohen’s *f* = 0.32, α = 0.05, power = 0.95), it was determined that a total sample of 24 participants would sufficiently detect significant effects. Accordingly, using the data from related published research (i.e., Ypsilanti et al., 2019, 2020) we calculated that in order to detect a large multivariate effect size (*f*^2^ = 0.58, α = 0.05, power = 0.95, with two predictor variables) in the multiple regression analysis, we needed a total sample of 25 participants.

All PTSD diagnosed veterans reported having a diagnosis of the condition preceding data collection by at least 12 months, with their respective diagnoses directly relating to varied theaters of conflict around the world; dating back to 1982 (The Falklands) and as recent as a few years ago (Afghanistan). The PTSD-diagnosed participants were recruited via networks that were established alongside various veteran PTSD support groups and care agencies. The research was carried out in accordance with the Code of Human Research Ethics of the British Psychological Society, and participants were provided with consent forms to complete, and were duly informed about their participation rights (i.e., voluntary and anonymous participation; no penalties for withdrawing from the study at any stage without notice).

### Measures

#### Demographics

Demographic characteristics were assessed with open-ended questions asking participants to indicate their age (i.e., How old are you?), gender, and nationality. War veterans were also asked to about their military background and service.

#### Posttraumatic Stress Disorder Measure

The PTSD Checklist for DSM-5 (PCL-5, Blevins et al., 2015) is a 20-question assessment tool of a person’s PTSD symptomatology, addressing each of the required Criteria B, C, D, and E from DSM-5 (American Psychiatric Association [APA], 2013). Questions are Likert scaled, ranging from zero (not at all) to four (extremely), such as (in the past month how much were you bothered by) “repeated, disturbing dreams of the stressful experience,” “trouble remembering important parts of the stressful experience,” and “feeling jumpy or easily startled.” Scores range from 0 to 80, a higher score indicative of a higher level of PTSD symptomatology, with a stipulated score of 33 suggested as a threshold for consideration of further assessment toward a potential PTSD diagnosis. In the present study, the PCL-5 had high internal consistency reliability (Cronbach’s α = 0.95).

#### Loneliness

The University of California Loneliness Scale (UCLA-3) was used in the present study (Russell, 1996) as a measure of loneliness, which consists of 20 items (e.g., “I am unhappy doing so many things alone,” “I feel completely alone,” and “I feel isolated from others”) rated on a 4-point scale of (1 = *never*, 4 = *often*). Loneliness scores are calculated by summing the items (after reverse scoring) and ranged from 20 to 80, with higher scores indicating increased loneliness levels. Cronbach’s alpha was high (α = 0.89).

#### Depression

Depressive symptomatology was measured using the Beck’s Depression Inventory (Beck et al., 1961), which measures characteristic attitudes and symptoms of depression and consists of 21 self-report items (e.g., “I feel sad,” “I feel discouraged about the future,” “I feel guilty all the time,” and “I am disgusted with myself”) rated on a 4-point scale (0–3, with variable anchors) giving a total score maximum of 63. Internal consistency reliability for the BDI in the present study Cronbach’s alpha was high (α = 0.91).

#### Self-Disgust

Self-disgust was assessed with the Self-Disgust Scale (SDS; Overton et al., 2008), an 18-item measure reflecting disgust and repulsion directed to the self. Six items are filler items (e.g., “I enjoy the company of others”) and 12 items reflect self-disgust toward the self (e.g., “I find myself repulsive”), and toward one’s behavior/actions (e.g., “I often do things I find revolting”). Responses are coded on a 7-point Likert scale (1 = *strongly agree*, 7 = *strongly disagree*), and, after reverse scoring 9 items, a total sum score is computed. Higher scores indicate higher levels of self-disgust. In the present study, the internal consistency reliability coefficient for the self-disgust scale was high (Cronbach’s α = 0.91).

#### Anxiety

The State/Trait Anxiety Inventory (STAI) (Spielberger, 1983) was used to measure trait anxiety in all participants. There are 10 Likert scale questions, graded from one (Not at all) to four (Very much so); such as “I feel tense,” “I feel nervous,” and “I feel steady.” Larger scores indicated a higher level of anxiety, and the internal consistency reliability coefficient in the present study was high (Cronbach’s α = 0.95).

### Eye-Tracking Task/Apparatus

A Tobii TX300 mobile eye tracker was used to record eye gaze for images of the self and other unknown faces. The Tobii TX300 provides high-quality fixation data through a non-invasive measure, allowing slight movements and comfort of the participants (Tobii, 2016). The stimulus created on Tobii Studio Software used 12 facial photographs (6 female, 6 male) showing neutral expressions from the Karolinska Directed Emotional Faces Database (Lundqvist et al., 1998). All photographs were cropped into an oval shape, removing hair, neckline and any other background stimulus that could also distract attention. Each oval-shaped face had the approximate dimensions of 401 × 578 mm. The presentation encompassed 48 slides, each presenting 2 faces alongside each other on a white background (Figure 5). Each slide was presented for 5 s and every slide separated with a control slide presenting a single fixation cross in the center of the screen. Half of these slides (24) included the participants’ own photograph as one of the faces displayed, also cropped in the same oval shape. The rest of the slides depicted two unknown faces. All slides were counterbalanced to ensure there was no preference shown to a particular side of the screen (left/right). Participants either saw slides presenting two unknown faces or one unknown face and their own face.

### Design/Procedure

A cross-sectional, correlational design was used to measure the associations between demographic characteristics (age, gender, and nationality), self-disgust, depressive symptomatology, loneliness, and anxiety in the two groups. No time restrictions were applied and survey completion required approximately 15–20 min. A mixed factorial design was used to investigate attentional vigilance (measured by time to first fixation in second 1) and attentional avoidance (measured by total visit duration across time blocks). The repeated measures factors were type of image (self vs. other) and time (2, 3, 4, 5 s). The between subjects factor was group (PTSD vs. non-PTSD).

Upon arrival participants were asked whether they were willing to have their photo taken for the purposes of the study and signed consent for participating in the study and having their photo taken. This information was not provided before arrival so as to avoid beautification preparations for the photograph. A single picture was taken of the face of each participant using a digital camera that was then cropped in an oval shape and sized as described in the apparatus section above. All pictures were taken from the same distance and in a neutral background. Participants were then asked to complete the demographic questionnaire and loneliness scale while the researcher cropped and uploaded their own photo in the Tobii eye-tracker (approximately 10 min). All participants completed the remaining questionnaires after the end of the eye-tracking study. Photographs were deleted from the digital camera (permanent deletion) in front of the participant at the end of the study, and participants were fully debriefed about the purposes of the study. They were also given the right to withdraw their data from the study up to 7 days after the data collection by giving them a unique identification code. Ethics approval for the study was granted by the respective Ethics Review Board of the host institution.

### Data Analysis

Multivariate analysis of variance was used to compare differences in self-disgust scores, loneliness, depression and anxiety between individuals with PTSD and non-PTSD participants. Hierarchical linear regression analysis models were used to test the hypothesized relationships between the constructs. Regression-based multiple mediation analysis with bootstrapping (Preacher and Hayes, 2008; Hayes and Preacher, 2014) was further used to assess the indirect association between loneliness, anxiety and depressive symptoms in the two groups, via self-disgust. Finally, repeated measures ANOVAs were used to examine differences in time to first fixation and total gaze duration in the two groups. All data were analyzed in SPSS v. 22 (IBM Corp, 2013) and Jamovi Version 0.9 (The Jamovi Project, 2019)^1^.

## Results

### Group Differences in Loneliness, Self-disgust, Anxiety, and Depression

To examine the first hypothesis, that PTSD veterans will exhibit higher levels of loneliness, self-disgust, and symptoms of depression and anxiety compared to non-PTSD participants, we performed a one-way between groups MANOVA with four dependent variables. Our results indicated a significant main effect of group on self-disgust, *F*(1,41) = 50.49, *p* = 0.000, η*_*p*_^2^* = 0.564; loneliness, *F*(1,41) = 48.27, *p* = 0.000, η*_*p*_^2^* = 0.553; depression *F*(1,41) = 112.18, *p* = 0.000, η*_*p*_^2^* = 0.742; and anxiety, *F*(1,41) = 41.16, *p* = 0.000, η*_*p*_^2^* = 0.514. In all variables war veterans with PTSD scored significantly higher than the comparison group with large effect sizes (Cohen, 1992) and substantially raised scores in the self-disgust scale. Descriptive statistics are presented in Table 1.

**TABLE 1:** Between group differences in self-disgust, loneliness, depression, and anxiety.

	PTSD veterans (*N* = 19)		Non-PTSD healthy controls (*N* = 22)	
	*M*	*SD*	*M*	*SD*
Self-disgust	55.36	12.66	28.27	11.73
Loneliness	58.68	8.44	36.09	11.79
Depression	32.00	9.62	5.72	6.08
Trait anxiety	30.10	5.51	18.04	6.38

### Direct Effects of Loneliness and Self-Disgust on Depression and Anxiety Symptoms

Four hierarchical linear regression models were used to assess the associations between loneliness and self-disgust with depression and anxiety symptoms in PTSD and non-PTSD participants respectively (Hypothesis 2). The models in each group were completed in two steps with the first step including loneliness, and the second step included self-disgust. Entering the predictor variables in this sequence allows us to examine the unique effects of self-disgust on anxiety and depression after taking into account the effect of loneliness. Additionally, having separate analyses for PTSD and non-PTSD participants allows us to examine if the patterns of associations observed between loneliness, self-disgust and anxiety and depression symptoms separately in the two groups. In all four models, tolerance levels between the predictor variables were high (>0.523), suggesting no multicollinearity. Additionally, the observed multivariate effect sizes (*f*^2^) were determined based on Cohen (1992) conventions, and ranged from 1.68 to 3.54, indicating large effects.

In the PTSD group, the results showed that a significant model (*F* = 16.16, *p* = 0.001) emerged predicting 62.8% (Adjusted *R*^2^) in depression symptoms. At the first step of the analysis, loneliness was significantly associated with depression symptoms (β = 0.818, *p* < 0.001), and this effect was also retained after adding self-disgust in the second step. Self-disgust was not significantly associated with depression symptoms in the PTSD group.

The second model in the PTSD group was also statistically significant (*F* = 16.60, *p* < 0.001) and predicted 63.4% (Adjusted *R*^2^) in anxiety symptoms. At the first step of the analysis, loneliness was significantly associated with anxiety symptoms (β = 0.758, *p* < 0.001). However, the effect of loneliness was reduced (β = 0.476, *p* = 0.02) after adding self-disgust, which was significantly associated with anxiety symptoms (β = 0.424, *p* = 0.04). Adding self-disgust in the final step of the analysis significantly increased predicted variance in anxiety symptoms (Δ*R*^2^ = 10%, *F*_change_ = 4.94, *p* = 0.04). The results from both regression models for the PTSD group are summarized in Table 2.

**TABLE 2:** Associations between loneliness, self-disgust, depression and anxiety in the PTSD group.

	Depression symptoms				Anxiety symptoms			
	*B*	β	95% CI for B	Adjusted *R*^2^	*B*	β	95% CI for B	Adjusted *R*^2^
Step 1				0.649				0.549
Loneliness	0.932	0.818**	0.597, 1.268		0.495	0.758**	0.277,0.713	
Step 2				0.628				0.634
Loneliness	0.929	0.815**	0.464, 1.394		0.311	0.476*	0.047,0.575	
Self-disgust	0.003	0.005	-0.307,0.313		0.185	0.424*	0.009,0.361	

Note: *p ≤ 0.05; **p ≤ 0.001.

In the non-PTSD participants, the analysis showed that a significant model emerged (*F* = 32.20, *p* < 0.001) predicting 74.8% (Adjusted *R*^2^) of the variance in depression symptoms. At the first step of the analysis, loneliness was significantly associated with depression symptoms (β = 0.822, *p* < 0.001). At the second step, self-disgust was entered and significantly increased predicted variance in depression symptoms (Δ*R*^2^ = 9.6%, *F*_change_ = 8.02, *p* = 0.01), was associated (β = 0.429, *p* = 0.01) with the criterion variable, and reduced the effect of loneliness (β = 0.526, *p* = 0.003).

The second model in the non-PTSD participants was also significant (*F* = 38.30, *p* < 0.001) and predicted 78% (Adjusted *R*^2^) of the variance in symptoms of anxiety. At the first step of the analysis, loneliness was significantly associated with anxiety symptoms (β = 0.821, *p* < 0.001). At the second step, self-disgust was entered in the analysis and significantly increased predicted variance in anxiety symptoms (Δ*R*^2^ = 12.7%, *F*_change_ = 12.14, *p* = 0.002), was associated (β = 0.493, *p* = 0.002) with the criterion variable, and reduced the effect of loneliness (β = 0.481, *p* = 0.003). The results from both regression models for the non-PTSD participants are presented in Table 3.

**TABLE 3:** Associations between loneliness, self-disgust, depression and anxiety in the non-PTSD group.

	Depression symptoms				Anxiety symptoms			
	*B*	β	95% CI for B	Adjusted *R*^2^	*B*	β	95% CI for B	Adjusted *R*^2^
Step 1				0.660				0.658
Loneliness	0.425	0.822***	0.287,0.562		0.445	0.821***	0.301,0.589	
Step 2				0.748				0.780
Loneliness	0.272	0.526**	0.108,0.435		0.260	0.481***	0.100,0.421	
Self-disgust	0.222	0.429*	0.058,0.387		0.268	0.493*	0.107,0.429	

Note: *p ≤ 0.05; **p ≤ 0.005; ***p ≤ 0.001.

### Indirect Effects of Loneliness on Depression and Anxiety Symptoms

**FIGURE 1:**
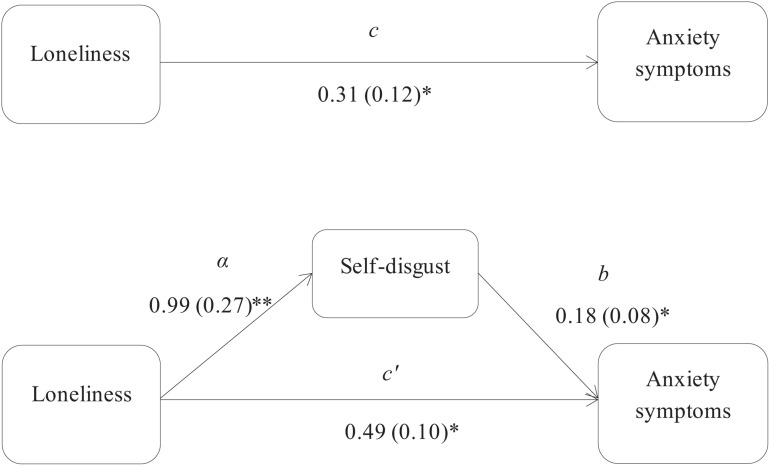
Self-disgust mediates the association between loneliness and anxiety symptoms in the PTSD group. *Note*: The total (c) and the indirect effect (c’) of loneliness on anxiety symptoms are shown; Unstandardized path coefficients are presented, with standard errors in brackets. ^∗^*p* ≤ 0.05; ^∗∗^*p* ≤ 0.005.

**FIGURE 2:**
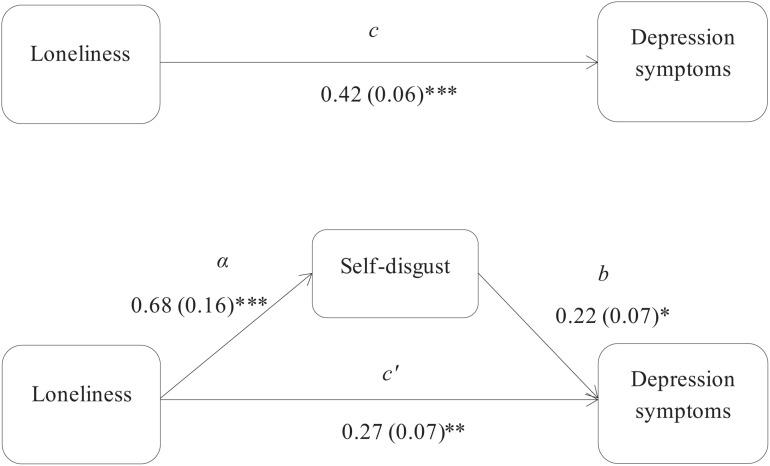
Self-disgust mediates the association between loneliness and depression symptoms in the non-PTSD group. *Note*: The total (c) and the indirect effect (c’) of loneliness on depression symptoms are shown. Unstandardized path coefficients are presented, with standard errors in brackets. ^∗^*p* ≤ 0.05; ^∗∗^*p* ≤ 0.005; ^∗∗∗^*p* ≤ 0.001.

**FIGURE 3:**
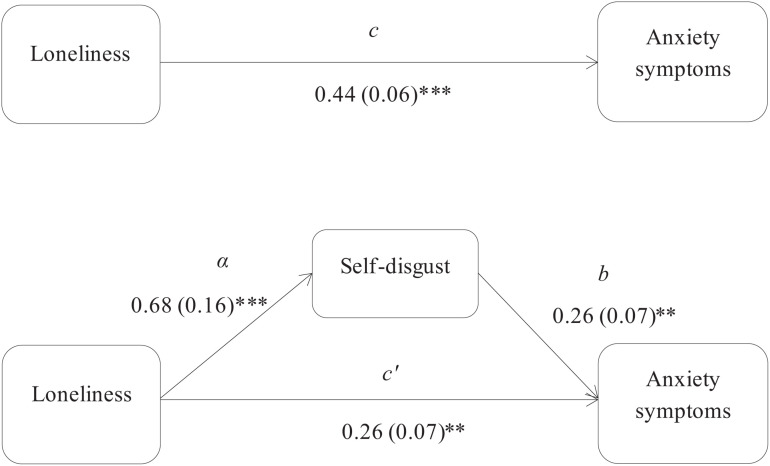
Self-disgust mediates the association between loneliness and anxiety symptoms in the non-PTSD group. *Note*: The total (c) and the indirect effect (c’) of loneliness on anxiety symptoms are shown. Unstandardized path coefficients are presented, with standard errors in brackets. ^∗^*p* ≤ 0.05; ^∗∗^*p* ≤ 0.005; ^∗∗∗^*p* ≤ 0.001.

Using the Preacher and Hayes (2008) approach, three mediation models were used to assess the indirect associations, via self-disgust, of loneliness with depression and anxiety symptoms in the PTSD and non-PTSD participants. Specifically, the first mediation model examined the indirect effect of loneliness on anxiety symptoms in the PTSD group, and showed that self-disgust had a significant mediation effect (*z* = 1.99, *p* = 0.04; Figure 1). The second and third models examined the indirect effects of loneliness, via self-disgust, on depression and anxiety symptoms respectively in the non-PTSD participants. The results showed that self-disgust mediated the association of loneliness with both depression (*z* = 2.46, *p* = 0.01 Figure 2) and anxiety symptoms (*z* = 2.80, *p* = 0.005 Figure 3).

### Eye-Tracking Data

Eye-tracking data were analyzed after determining the areas of interest (AoI) in Tobii studio. AoI were the persons’ own face in slides that depicted the “self” alongside a face of an unknown other. Then we defined AoI the “other” face in slides that depicted the “self” alongside a face of an unknown other. We extracted two metrics for these AoI, time to first fixation (TFF) and total visit duration (TVD). In the final analysis we compared TFF and TVD toward the “self” picture and the “other” picture in both groups. A 2 × 2 repeated measures ANOVA was used to assess attentional vigilance from differences in time to first fixation toward their own picture and the picture of an unknown other between the two groups (PTSD vs. non-PTSD) for each Type of Stimulus (self vs. other). There was no significant main effect of group, *F*(1,39) = 0.98, *p* > 0.05, or Type of Stimulus, *F*(1,39) = 2.66, *p* > 0.05, and there was no significant interaction, *F*(1,39) = 0.47, *p* > 0.05. This means that there were no differences in attentional vigilance between the two groups when looking at their own face and the faces of unknown others. To examine attentional avoidance we used a 2 × 2 × 4 repeated measures ANOVA with group (PTSD, non-PTSD) as a between subject factor and Type of Stimulus (picture of self vs. picture of unknown other) and time blocks (2 seconds, 3 seconds, 4 seconds and 5 seconds) as repeated measures factors and compared total eye gaze duration in each second. There was a significant main effect of Time Blocks, *F*(3,39) = 5.56, *p* = 0.001, η*_*p*_^2^* = 0.12, and a significant interaction between Type of Stimulus × Group, *F*(1,39) = 4.91, *p* = 0.03, η*_*p*_^2^* = 0.11. Means and SDs of each group across 4 time blocks are presented in Table 4.

**TABLE 4:** Means and SDs of total eye gaze (measured by total visit duration) to the picture of the self and the picture of an unknown other in PTSD and non-PTSD groups across time blocks.

	PTSD veterans (*N* = 19)		Non-PTSD healthy controls (*N* = 22)	
	*M*	*SD*	*M*	*SD*
Total visit duration to self (second 2)	0.50	0.16	0.55	0.15
Total visit duration to self (second 3)	0.55	0.17	0.64	0.13
Total visit duration to self (second 4)	0.58	0.17	0.64	0.12
Total visit duration to self (second 5)	0.58	0.13	0.59	0.15
Total visit duration to other (second 2)	0.64	0.13	0.58	0.12
Total visit duration to other (second 3)	0.67	0.14	0.59	0.12
Total visit duration to other (second 4)	0.67	0.13	0.58	0.15
Total visit duration to other (second 5)	0.68	0.12	0.57	0.10

**FIGURE 4:**
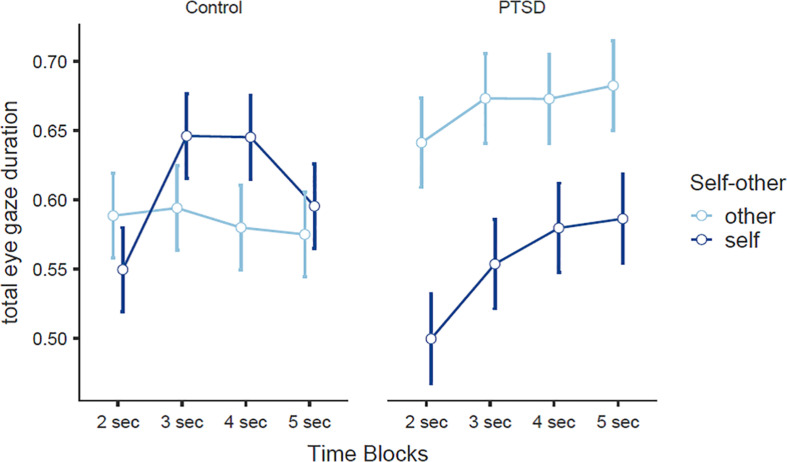
Total eye gaze duration across 4 time blocks in war veterans with PTSD and non-PTSD individuals for their own face and the face of an unknown other.

We further explored the avoidance hypothesis using 2 × 4 repeated measures ANOVAs for each group separately. For the non-PTSD participants there was a significant main effect of Time Block, *F*(3,21) = 3.87, *p* = 0.013, η*_*p*_^2^* = 0.15, and a significant interaction Type of Stimulus and Time Block, *F*(3,63) = 2.96, *p* = 0.039, η*_*p*_^2^* = 0.12 (Figure 4). Planned *post hoc* comparisons indicated a significant increase in total eye gaze toward their own face from second 2 to second 3, *t* = 4.61, *p* < 0.001, df = 21, and second 4, *t* = 3.25, *p* = 0.004, df = 21. No other differences were significant. For the PTSD group there was a significant main effect of Time Block, *F*(3,18) = 3.23, *p* = 0.029, η*_*p*_^2^* = 0.15, and a significant main effect of Type of Stimulus, *F*(1,18) = 6.46, *p* = 0.02, η*_*p*_^2^* = 0.26 (Figure 4), with PTSD participants spending more time looking at other unknown faces compared to their own face (measured as total eye gaze duration) (other unknown faces M = 0.66, SE = 0.025, own face M = 0.55, SE = 0.028). The interaction was not significant in this group. Planned *post hoc* comparisons indicated that PTSD participants spend less time looking at themselves in second 2 (*t* = 2.38, *p* < 0.02, df = 18), second 3 (*t* = 2.08, *p* = 0.05, df = 18), and second 5 (*t* = 2.49, *p* = 0.02, df = 18).

**FIGURE 5:**
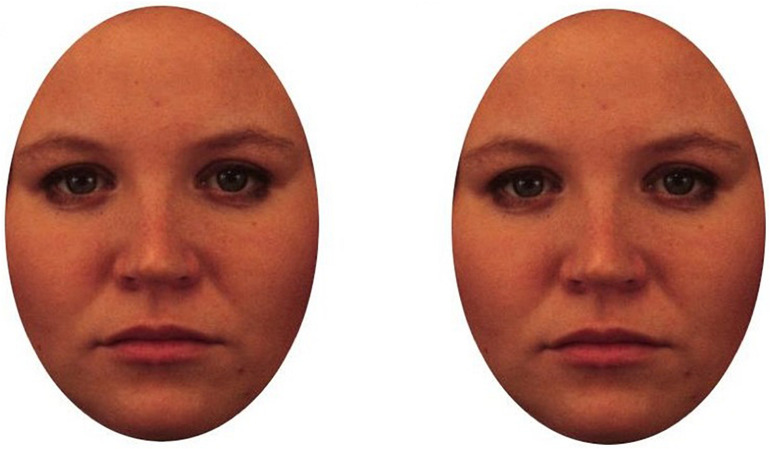
Sample of the eye-tracking stimuli from the Karolinska database faces depicting two unknown faces.

Finally, to explore hypothesis 4, that there will be a correlation between total eye gaze duration for the self/others and trait self-disgust, we calculated the difference between total eye gaze toward other unknown faces and toward the self (mean other–mean self) and correlated this difference with self-disgust scores. We found a significant positive correlation (Pearson’s) *r* = 0.31, *p* < 0.05 suggesting that as self-disgust scores increase the mean total eye gaze away from the self and toward other unknown faces also increases. This finding provides additional evidence for the avoidance hypothesis of self-disgust.

## Discussion

In the present study we investigated, for the first time, self-disgust, loneliness and mental health outcomes in war veterans with PTSD. We also explored the mediating role of self-disgust in the relationship between loneliness and depression and anxiety in veterans with PTSD and non-PTSD individuals from the general population. We hypothesized that war veterans with PTSD would experience higher levels of loneliness, self-disgust, anxiety, and depression compared to the general population. Our findings supported this hypothesis. Specifically, war veterans with PTSD reported higher scores in all the examined variables with large effect sizes following Cohen’s conventions (Cohen, 1969; Richardson, 2011). Our second hypothesis stated that self-disgust will mediate the relationship between loneliness and symptoms of anxiety and depression in both groups (Ypsilanti et al., 2019, 2020). This hypothesis was partially supported by our findings. Specifically, in war veterans with PTSD, self-disgust significantly improved predicted variance in anxiety symptoms, over and above the effects of loneliness, and also mediated the relationship between loneliness and anxiety. However, self-disgust did not increase predicted variance in depression symptoms and did not mediate the association between loneliness and depression in war veterans with PTSD. In the non-PTSD participants, self-disgust significantly increased predicted variance in both anxiety and depression symptoms, over and above the effects of loneliness, and significantly mediated the association of loneliness with both depression and anxiety symptoms.

Our findings are largely in support of previous research where self-disgust mediated the association between loneliness and symptoms of depression in the general population (Ypsilanti et al., 2019). The present study also further extends previous research on self-disgust and mental health difficulties (e.g., Brake et al., 2017; Clarke et al., 2019) by showing that self-disgust mediated the association between loneliness and anxiety symptoms in both war veterans with PTSD and a non-PTSD group from the general population. However, the non-significant mediation effect of self-disgust on the loneliness-depression association in the PTSD group is in contrast with previous research where self-disgust prospectively predicted depression (Powell et al., 2013), mediated the relationship between dysfunctional thoughts and depression (Overton et al., 2008), and mediated the relationship between loneliness and depression in the general population (Ypsilanti et al., 2019).

Previous research has also suggested that loneliness induces negative affective states and the tendency to misinterpret social contact as threatening (i.e., social threats), which may enhance social withdrawal and increase negative ruminations toward social interactions (Cacioppo and Hawkley, 2009; Hawkley and Cacioppo, 2010; Cacioppo et al., 2015). Although this has not been directly explored in war veterans with PTSD it is possible that loneliness in this population increases anxiety by creating a negative affectivity loop via self-disgust. This means that rather than experiencing depressive symptoms due to self-disgust (an effect observed in the general population; Overton et al., 2008; Powell et al., 2013), war veterans with PTSD may experience anxiety-related symptoms stemming from negative self-directed emotions. Based on the findings from the present study, we suggest that self-disgust may represent an affective mechanism through which loneliness progresses to the development of anxiety in war veterans with PTSD, but future research is needed to empirically examine this process.

Importantly, in the present study we investigated for the first time the avoidance hypothesis of self-disgust (Powell et al., 2013) in war veterans with PTSD using eye-tracking methodology. For this purpose, we developed a novel task that exposed participants to a picture of their own face alongside a picture of the face of an unknown other. We used time to first fixation as an index of attentional vigilance and found no differences between the two groups (i.e., veterans with PTSD and healthy controls). We used total visit duration across time blocks (2 s, 3 s, 4 s, and 5 s) to examine attentional avoidance following previous research (Armstrong and Olatunji, 2012; Ypsilanti et al., 2020). Our results demonstrated that war veterans with PTSD behave differently when exposed to such stimuli compared to a group of people from the general population. Specifically, veterans with PTSD spent much more time gazing at the faces of unknown other vs. their own face, than people without PTSD. Moreover, this gaze pattern (attentional avoidance) is initiated after the 3^rd^ second of stimulus exposure—as Figure 4 shows, there is a sharp drop in eye-gaze after 3 s. On the contrary, non-PTSD participants gazed at their own image more time compared to the picture of an unknown other, and did not exhibit avoidance to the self. Specifically, while there is a relatively stable eye-gaze pattern for the image of the unknown other, there is temporarily an increased gaze time at 3 s and 4 s to the image of the self. Taken together, our study shows that veterans with PTSD reported almost three times higher scores in self-disgust, and tended to visually avoid pictures of themselves (vs. others) at later exposure times. Further, the significant positive correlation between total visit duration and self-disgust scores in PTSD participants (Hypothesis 4) suggests that this eye-gaze pattern is, at least partly, related to higher self-disgust scores. Based on previous reports, individuals with high self-disgust avoid their own reflection and direct their eye gaze away for their own image (Ypsilanti et al., 2020). One possible explanation for this pattern of eye-gaze in veterans with PTSD is that their image may serve as a reminder of a negative “self” that triggers dysphoria and therefore, avoidance acts as a coping mechanism to relief this negative self-perception. In the present study, we used for the first time an eye-tracking task that exposed participants to their own image alongside another unknown face. By doing so, we were able to determine whether eye gaze was directed to the periphery of their own face (outside the area of interest) or toward the face of an unknown other (inside another area of interest) that does not trigger dysphoric emotions. We found that veterans with PTSD preferred to gaze at other people’s faces when they avoided their own image rather than look away from faces altogether. Since this is the first time that eye-gaze has been recorded in such an eye-tracking paradigm among veterans with PTSD conclusions should be drawn with caution.

### Strengths and Limitations

Our study has both theoretical and methodological strengths. In terms of theory, we identified a previously unnoticed variable (i.e., self-disgust) that can potentially explain the association between loneliness and anxiety symptoms. This is important for the following reasons. Previous research has shown that self-disgust is implicated in survivors of traumatic experiences (for a review see Clarke et al., 2019) but most of this research dealt with sexual abuse-related trauma, whereas our study is the first to examine self-disgust in combat-related PTSD among veterans. Secondly, our study highlighted the positive association between self-disgust and loneliness, which was found to be stronger among veterans with PTSD than in the general population. Accordingly, our results further emphasized the mediating properties of self-disgust in the association between loneliness and anxiety symptoms in veterans with PTSD—this advances recent research that reported similar findings in older adults (Ypsilanti et al., 2020).

Methodologically, one of the main strengths of the current study is the application of a novel psychophysiological paradigm to attentional vigilance and avoidance in war veterans diagnosed with PTSD - a population that is difficult to access and recruit in basic psychological research. Eye-tracking tasks provide a unique opportunity to identify early and later attentional processing by allowing individuals to naturally dwell of the stimuli. This novel task exposed participants to their own face alongside an unknown face, to explore whether avoidance would take place (Powell et al., 2013). Presenting two faces simultaneously on the screen allowed us to determine whether eye gaze avoidance was detected in the periphery of their own face or whether there was a more conscious preference to gaze at an unknown other. Another strength of our study is the counterbalanced order of the eye-tracking task and the completion of the questionnaires. This was done to avoid any priming effects that the questionnaires would have on the eye-tracking task.

We also acknowledge several limitations that could be overcome in future research. Firstly, the cross-sectional design limits our potential to make causal inferences about the association between loneliness, self-disgust, and mental health outcomes in PTSD and non-PTSD adults. Longitudinal studies could potentially determine the temporal association between these variables and indicate whether loneliness precedes self-disgust in the genesis of anxiety and depression. It is also important to note that in the present study we did not use war veterans without PTSD as a control group against war veterans with PTSD. Given that our intention was to replicate the mediating role of self-disgust on the associations of loneliness with depression and anxiety symptoms in the general population that was recently reported in the literature (Ypsilanti et al., 2019, 2020), we selected participants without PTSD from the general population as a comparison group.

## Data Availability Statement

The raw data supporting the conclusions of this article will be made available by the authors, without undue reservation.

## Ethics Statement

The studies involving human participants were reviewed and approved by the Sheffield Hallam University Ethics Review Committee. The patients/participants provided their written informed consent to participate in this study.

## Author Contributions

AY conceived the idea of the study, contributed to the design and data analysis, and authored the manuscript. RG designed the tasks, recruited and collected all the data, and contributed to that data analysis. LL contributed to the data analysis and co-authored the manuscript. AR contributed to the design of the task and data analysis. PP contributed to the data analysis and edited the revisions of the manuscript. PO contributed to the data analysis and edited the revisions of the manuscript. All authors contributed to the article and approved the submitted version.

## Conflict of Interest

The authors declare that the research was conducted in the absence of any commercial or financial relationships that could be construed as a potential conflict of interest.

## Footnotes

1 Retrieved from https://www.jamovi.org
